# The stoichiometric divisome: a hypothesis

**DOI:** 10.3389/fmicb.2015.00455

**Published:** 2015-05-12

**Authors:** Alexander J. F. Egan, Waldemar Vollmer

**Affiliations:** The Centre for Bacterial Cell Biology, Institute for Cell and Molecular Biosciences, Newcastle University, Newcastle upon Tyne, UK

**Keywords:** bacterial cell division, peptidoglycan, peptidoglycan synthesis, divisome and multiprotein complex

## Abstract

Dividing *Escherichia coli* cells simultaneously constrict the inner membrane, peptidoglycan layer, and outer membrane to synthesize the new poles of the daughter cells. For this, more than 30 proteins localize to mid-cell where they form a large, ring-like assembly, the divisome, facilitating division. Although the precise function of most divisome proteins is unknown, it became apparent in recent years that dynamic protein–protein interactions are essential for divisome assembly and function. However, little is known about the nature of the interactions involved and the stoichiometry of the proteins within the divisome. A recent study (Li et al., 2014) used ribosome profiling to measure the absolute protein synthesis rates in *E. coli*. Interestingly, they observed that most proteins which participate in known multiprotein complexes are synthesized proportional to their stoichiometry. Based on this principle we present a hypothesis for the stoichiometry of the core of the divisome, taking into account known protein–protein interactions. From this hypothesis we infer a possible mechanism for peptidoglycan synthesis during division.

## Introduction

The peptidoglycan cell wall is an essential component of most bacteria, required for the maintenance of cell morphology and structural integrity (Weidel and Pelzer, 1964). Peptidoglycan (PG) forms a net-like, continuous layer, called the sacculus, surrounding the cytoplasmic membrane. Thus, a bacterial cell needs to increase the surface of its sacculus in order to grow and divide. Sacculus growth has to be well-controlled, because the accumulation of defects may lead to cell lysis; this is the case when antibiotics like β-lactams inhibit peptidoglycan synthesis. Remarkably, the growth of the single-layered sacculus in *E. coli* is accompanied by the release of as much as 50% of the total peptidoglycan material per generation by hydrolases (Goodell, 1985). What are the molecular mechanisms of sacculus growth? Höltje (1998) proposed that multi-enzyme complexes made of peptidoglycan synthases and hydrolases simultaneously synthesize new peptidoglycan and incorporate it into the sacculus, and remove old material, by a so-called 3-for-1 mechanism. Subsequent experimental evidence supported the multi-enzyme complex hypothesis. Genetic and biochemical data have demonstrated many protein–protein interactions between various peptidoglycan synthases, hydrolases, regulatory proteins, and cytoskeletal elements (reviewed in Typas et al., 2012; Egan and Vollmer, 2013). These have been observed in several species including, but not limited to, the Gram-positive *Bacillus subtilis*, *Staphylococcus aureus* and *Streptococcus pneumoniae* and the Gram-negative *Caulobacter crescentus* and *E. coli*, which will be the focus of this article.

In *E. coli*, cell division involves over 30 proteins, with twelve of these (the Fts proteins and ZipA) absolutely required for the process (Margolin, 2005). The assembly of essential proteins occurs in two steps; FtsZ, FtsA, ZipA, Zap proteins (A–E) and FtsEX assemble early at the future division site, before any constriction is visible. Immediately before the onset of constriction the divisome matures through the incorporation of FtsK, FtsQ, FtsL, FtsB, FtsW, PBP3 (FtsI), and FtsN (Aarsman et al., 2005). By a largely unknown process these proteins, along with other accessory proteins, then facilitate the synthesis of the new cell poles of each daughter cell (reviewed in Typas et al., 2012; Egan and Vollmer, 2013). Although different methodologies have identified a large number of interactions between divisome proteins (Egan and Vollmer, 2013), for most of these the precise interaction sites are not known. Moreover, while the cellular copy number of some but not all divisome proteins have been reported over the years, the stoichiometry of proteins within the divisome is not known.

A recent study by Li et al. (2014) used ribosome profiling/footprinting to evaluate the genome-wide absolute protein synthesis rates and protein copy numbers in *E. coli*. Interestingly, they observed proportional synthesis of proteins present in multiprotein complexes. Proteins of 59 out of 64 cytosolic and membrane complexes (92%) with known stoichiometry were found to be synthesized proportionally to their stoichiometry. Based on this principle of proportional synthesis of proteins participating in complexes, and using the protein synthesis rates from Li et al. (2014), we suggest a hypothetical model for the core divisome complex in *E. coli* factoring in known protein–protein interactions. From this hypothetical stoichiometry model we suggest possible aspects of the mechanisms for PG synthesis during division.

## Using the Ratios of Division Proteins to Suggest a Stoichiometry for the Complex

Our aim here was to model the complex formed by the late division proteins and FtsA, without FtsZ and other cytoplasmic or accessory components. PBP3 is the monofunctional peptidoglycan transpeptidase (TPase) essential for cell division in *E. coli* (Weiss et al., 1997), and there is no evidence that PBP3 is active elsewhere in the cell other than the division site. We therefore surmised PBP3 is a reasonable choice for use as the reference point for our considerations on divisome stoichiometry. To this end we calculated the ratios of absolute synthesis rates of each of the *E. coli* cell division proteins (FtsA, FtsK, FtsQ, FtsL, FtsB, PBP3 (*ftsI*), FtsW, FtsN, and PBP1B; Li et al., 2014) to the synthesis rate of PBP3 for cells grown in both minimal and complete media (MOPS media with either full or minimal supplement; Table 1). The ratios are similar at both conditions except for PBP1B and its interacting protein FtsN (Müller et al., 2007) which are both slightly more abundant in complete media for unknown reason. This increased abundance may reflect the fact that PBP1B is able to function outside of the divisome, presumably in cell elongation. PBP1B has been shown to be functionally redundant with the other major PG synthase of *E. coli*, PBP1A (Yousif et al., 1985; Denome et al., 1999). FtsN interacts with PBP1B in non-dividing cells (Müller et al., 2007) and hence might be associated with PBP1B at all times. An apparent disparity in synthesis rate was seen with the OM lipoprotein regulator of PBP1B, LpoB, which is in sixfold or fourfold excess over its cognate synthase in minimal and compete media, respectively. It was previously shown that PBP1B and LpoB proteins interact with a 1:1 stoichiometry (Egan et al., 2014). The reason for the excess of LpoB is unknown, but may be of regulatory consequence. LpoB is absolutely essential for PBP1B function in the cell (Paradis-Bleau et al., 2010; Typas et al., 2010), therefore, an excess of LpoB may increase the likelihood that PBP1B can be activated. However, we cannot exclude other mechanisms including, for example, enhanced turnover of LpoB in the cell, necessitating higher synthesis.

**TABLE 1:** **Synthesis rates of the late division proteins (and FtsA) and their ratios relative to PBP3 in MOPS minimal and complete media according to Li et al. (2014)**.

	**MOPS minimal**			**MOPS complete**		
**Protein**	**Copy number**	**∼ Ratio to PBP3**	**Molecules/complex^a^**	**Copy number**	**∼ Ratio to PBP3**	**Molecules/complex^a^**
PBP3	144	1.0	2	349	1.0	2
PBP1B	139	1.0	2	512	1.5	2/3
LpoB	954	6.6	2^b^	1490	4.3	2^b^
FtsN	269	1.9	4	871	2.5	4/5
FtsW	117	0.8	2	293	0.8	2
FtsQ	147	1.0	2	336	1.0	2
FtsL	201	1.4	2	416	1.2	2
FtsB	140	1.0	2	487	1.4	2
FtsK	213	1.5	3	508	1.5	3
FtsA	575	4.0	8	984	2.8	6

^a^We have assumed that previously observed homodimerizations (e.g., PBP3, PBP1B) occur within the divisome.

^b^Despite the apparent excess of LpoB, we have assumed two molecules per complex given its stoichiometry with PBP1B is 1:1 (Egan et al., 2014).

PBP3 interacts with PBP1B and both form, and are likely functional as, homodimers (Zijderveld et al., 1991; Bertsche et al., 2005, 2006; Sauvage et al., 2014). We therefore assume that there are two molecules of PBP3 and two molecules of PBP1B present in a functional peptidoglycan synthesis unit (or complex). Interestingly, most other late division proteins (except FtsK, FtsL, and FtsN) had ratios of approximately 1:1 with PBP3 and PBP1B. FtsL, whose ratio to PBP3 is 1.4:1, is part of the FtsQLB complex within the divisome (Buddelmeijer and Beckwith, 2004). FtsL is unstable in the absence of FtsB in both *B. subtilis* and *E. coli* (Daniel and Errington, 2000; Buddelmeijer et al., 2002), and FtsQ, FtsL, and FtsB appear to interact in equimolar stoichiometry; different models suggest a 1:1:1 or a 2:2:2 complex (Masson et al., 2009; Villanelo et al., 2011). We therefore assume two molecules FtsL per complex, equivalent to FtsQ and FtsB.

FtsN has been previously shown to self-associate (Di Lallo et al., 1999), but there are no published data on the stoichiometry of its interactions with PBP1B and PBP3 (Wissel and Weiss, 2004; Müller et al., 2007). Given its ratio of 2:1 to PBP3 (and PBP1B) FtsN may exist in the divisome as a tetramer, or as two separate dimers. FtsN is the last essential protein recruited to mid-cell, and its absence leads to the delocalization of the already assembled divisome components (Rico et al., 2010). Recent evidence suggests that FtsN is the ultimate regulator of septal PG synthesis. In addition to its interactions with PBP3 and PBP1B, FtsN interacts directly with the early divisome protein FtsA (Busiek et al., 2012). This interaction is thought to provide the final signal for the beginning of constrictive PG synthesis through both FtsA and the FtsQLB complex. It was recently shown that point mutations in *ftsA*, *ftsL*, and *ftsB* can bypass the need for FtsN and the altered proteins acted synergistically to restore cell division in the absence of FtsN, suggesting that FtsN signals for constriction via these proteins (Liu et al., 2015; Weiss, 2015). Of note, the copy number of ∼4500 FtsN molecules per cell determined previously by immunodetection (Ursinus et al., 2004) is significantly higher than the 260 (MOPS minimal)/870 (MOPS complete) molecules per cell determined by ribosome profiling (Li et al., 2014), suggesting that FtsN numbers might vary with strain and growth conditions, which were different in both studies.

FtsK is the first of the “late” divisome proteins, bridging the PG synthetic and cytoskeletal parts of the complex through interactions with FtsA and FtsQ (Chen and Beckwith, 2001; Buddelmeijer and Beckwith, 2004; Aarsman et al., 2005). FtsK has two domains, an N-terminal domain anchored in the inner membrane (IM) which is essential for cell division (Grenga et al., 2008; Dubarry et al., 2010) and a cytoplasmic C-terminal domain which hexamerizes to form a directional DNA pump to resolve chromosome dimers, which is essential in cells with catenated sister chromosomes (Aussel et al., 2002). These domains are linked by a 600 residue long flexible region (Löwe et al., 2008). A specific mutation in *ftsA* or overproduction of *ftsQ, ftsA*, and *ftsZ* can partially compensate for the loss of *ftsK*, suggesting that the essential role of FtsK in cell division may be ensuring divisome stability or spatial regulation (Geissler and Margolin, 2005). The N-terminal domain of FtsK was shown to form hexamers independently of the C-terminal domain and exist as such at midcell (Bisicchia et al., 2013). The mid-cell of predivisional or dividing cells contained 1–8 (average of 7) hexamers of FtsK (Bisicchia et al., 2013). The ratio of FtsK to PBP3 is 1.5:1, suggesting that there are three molecules of FtsK for every two of PBP3 and thus three FtsK molecules per complex. Therefore, the FtsK homohexamer appears to interact with two complexes. We therefore suggest that FtsK spatially co-ordinates two of the peptidoglycan synthesis complexes, which we term “synthesis nodes,” with the FtsZ-FtsA cytoskeletal structures in the cytoplasm.

**FIGURE 1:**
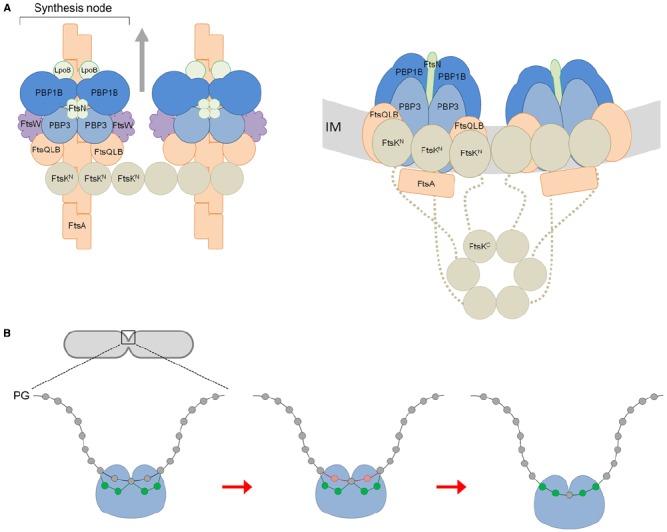
**A model for the divisome stoichiometry and its potential mechanism of action. (A)** Proposed divisome complex with stoichiometry according to protein synthesis rates. Left: view from above, without the inner membrane (IM). Right: side view, including the C-terminal domain of FtsK and flexible linker region (dashed line). Proteins are represented as colored spheres/ovals; synthases (PBP1B and PBP3) in blue, regulators (LpoB, FtsN) in green, lipid II flippase (FtsW) in purple, and other core proteins in orange (FtsQLB, FtsA) or brown (FtsK). Proteins are shown roughly to scale with known protein–protein interactions accommodated (summarized in Egan and Vollmer, 2013). In this snap-shot of the dynamic machinery two PG synthesis nodes are co-ordinated with FtsA filaments and an FtsK hexamer. We have not attempted to reconcile FtsZ, ZipA, or Zap proteins for simplicity. The arrows indicate the direction of complex movement and glycan chain synthesis. **(B)** View of PG synthesis from the perpendicular axis of the cell. The divisome complex, featuring two synthesis nodes coordinated by FtsK and the cytoskeletal proteins is shown as a single entity for simplicity (blue ball). Each synthesis node produces and cross-links two new glycan strands to the existing sacculus either side of a central strand. These four new glycans (shown in green) are synthesized and attached beneath two existing (docking) strands (shown in red) adjacent to the this central strand, which are simultaneously removed through the action of PG hydrolases as first proposed by Höltje (1998).

Figure 1A shows our hypothetical model for core divisome stoichiometry based on the determined protein ratios. The protein ratios suggest that there are two peptidoglycan synthesis nodes associated with an FtsK hexamer, when three may well be able to associate. The complex is likely highly dynamic, coalescing and dissociating repeatedly in the cell for separate rounds of PG synthesis. Remarkably, such a complex has a total of 42 membrane proteins together containing as many as 92 transmembrane helices. Hence, the impact of these on membrane properties is likely to be profound. Moreover, the divisome complex is likely to be even larger because we have not included accessory divisome components such as FtsEX, FtsP, ZipA or the peptidoglycan hydrolases.

## Constrictive PG Synthesis by the Divisome

At given conditions the cell maintains a constant diameter during growth, prior to division (Koch, 1995; Höltje, 1998). Presumably, the elongasome complex operates to robustly maintain cell diameter and rod-shape. In contrast, the divisome employs a constrictive mode of PG synthesis during cell division to produce the new cell poles, altering the architecture of the cell envelope. There are parallels between the divisome and elongasome in terms of their constituent proteins (Typas et al., 2012), and it was recently suggested that the divisome has evolved from the elongasome (Szwedziak and Löwe, 2013). Both contain class A and class B PBPs, a SEDS protein implicated in lipid II flippase activity (Mohammadi et al., 2011) and IM proteins which are likely required for spatial coordination and proper complex assembly. FtsK and FtsQLB are key components of the divisome with no known analog in the elongasome. They could be in part responsible for mediating constrictive peptidoglycan synthesis, ultimately driven by FtsZ dynamics.

After modeling a potential stoichiometry of the divisome we attempted to reconcile how the peptidoglycan machinery may function. According to our stoichiometry model the whole complex with two nodes would theoretically be capable of synthesizing four glycan chains and it has eight transpeptidase active sites. The latter can link the new glycan chains with each other and attach them to the existing sacculus. Given that the cell produces two identical new cell poles for each daughter we extend Höltje’s (1998) three-for-one model for the divisome. We suggest that each synthesis node works to produce and attach two new strands at two docking strands situated either side of another strand, coordinated spatially by FtsK and ultimately the cytoskeletal proteins. As with Höltje’s (1998) model the hydrolysis of the docking strands allows for the insertion of the new strands into the existing layer, to progress the septum closure. Indeed, elegant labeling experiments showed that 30–50% of the newly synthesized PG is removed simultaneously or shortly after it is incorporated into the new septum (Uehara and Park, 2008). The insertion of new material and the inward growth of the septum occurs in a symmetrical pattern (Figure 1B). This mechanism also accounts for the aberrant septation observed in certain peptidoglycan hydrolase mutants. *E. coli* cells deficient in *N*-acetylmuramyl-L-alanine amidases undergo cytokinesis and form septal PG between two daughter cells, but are unable to separate from each other due to the defect in cleavage of this septal PG (Heidrich et al., 2001). This observation can be explained by successive deposition of new septal PG without removal of the docking strands.

What drives constrictive peptidoglycan synthesis during division? A current model is that the FtsZ cytoskeletal ring, the assembly of which represents the first stage in the division process (Bi and Lutkenhaus, 1991), exerts a constrictive force on the IM under consumption of guanosine triphosphate (GTP) (reviewed in Erickson et al., 2010; Meier and Goley, 2014). In support of this a membrane-anchored version of FtsZ alone was able to produce visible invaginations in tubular unilaminar vesicles in the presence of GTP, but it could not produce sufficient force for full constriction (Osawa et al., 2009). It is thought that the switch of FtsZ filaments from straight to curved conformations is the basis for constriction (Erickson et al., 2010). However, calculations based on the structural models of FtsZ filaments estimate the minimum diameter the Z-ring could achieve is between 50 and 250 nm, when considering additional factors such as the structures of FtsZ’s membrane anchors FtsA and ZipA. Neither diameter would allow for complete scission of the cell (Erickson et al., 2010). Thus, it was suggested that PG synthesis may contribute to the constrictive force in the later stages of cytokinesis (Joseleau-Petit et al., 2007; Erickson et al., 2010). This is consistent with the fact that efficient constriction only begins after the divisome has matured through the recruitment of FtsK and the other late division proteins (Aarsman et al., 2005), but is hardly occurring when the fully assembled FtsZ ring is still associated with the elongasome during pre-septal PG synthesis (de Pedro et al., 1997; Typas et al., 2012; van der Ploeg et al., 2013). The question of how PG synthesis by the divisome may contribute to constriction remains unclear. However, it is a reasonable assumption that the expansion of the septal PG due to the incorporation of new material at the tip of the inward growing septum exerts some force on other parts of the cell envelope.

In summary, we have modeled the stoichiometry of the complex responsible for peptidoglycan synthesis during division based on the average copy numbers of the core proteins in the cell and known interactions. We have assumed that the synthesis of the proteins within the complex is proportional, as observed in ∼90% of known complexes of *E. coli*. However, we are aware that in the cell the divisome is likely to be highly dynamic, thus our model represents a single view of one of the core complexes, presumably its final assembly state. From this model we propose an update to Höltje’s (1998) three-for-one model with regard to the functioning divisome, such that four strands are simultaneously incorporated while two are removed. We expect that our hypothetical model will be tested and improved in the coming years, as our understanding of the divisome’s constituent proteins deepens.

### Conflict of Interest Statement

The authors declare that the research was conducted in the absence of any commercial or financial relationships that could be construed as a potential conflict of interest.
